# Chromosome-level genome assembly and structural variant analysis of two laboratory yeast strains from the Peterhof Genetic Collection lineage

**DOI:** 10.1093/g3journal/jkab029

**Published:** 2021-02-05

**Authors:** Yury A Barbitoff, Andrew G Matveenko, Anton B Matiiv, Evgeniia M Maksiutenko, Svetlana E Moskalenko, Polina B Drozdova, Dmitrii E Polev, Alexandra Y Beliavskaia, Lavrentii G Danilov, Alexander V Predeus, Galina A Zhouravleva

**Affiliations:** 1 Department of Genetics and Biotechnology, St. Petersburg State University, St. Petersburg 199034, Russia; 2 Bioinformatics Institute, St. Petersburg 197342, Russia; 3 St. Petersburg Branch, Vavilov Institute of General Genetics of the Russian Academy of Sciences, St. Petersburg 199034, Russia; 4 Irkutsk State University, Irkutsk 630003, Russia; 5 CerbaLab Ltd., St. Petersburg 199106, Russia; 6 Department of Invertebrate Zoology, St. Petersburg State University, 199034 St. Petersburg, Russia; 7 University of Liverpool, Liverpool, UK, L7 3EA

**Keywords:** Yeast genome, 74-D694, long reads, structural variant, *FLO* genes

## Abstract

Thousands of yeast genomes have been sequenced with both traditional and long-read technologies, and multiple observations about modes of genome evolution for both wild and laboratory strains have been drawn from these sequences. In our study, we applied Oxford Nanopore and Illumina technologies to assemble complete genomes of two widely used members of a distinct laboratory yeast lineage, the Peterhof Genetic Collection (PGC), and investigate the structural features of these genomes including transposable element content, copy number alterations, and structural rearrangements. We identified numerous notable structural differences between genomes of PGC strains and the reference S288C strain. We discovered a substantial enrichment of mid-length insertions and deletions within repetitive coding sequences, such as in the *SCH9* gene or the *NUP100* gene, with possible impact of these variants on protein amyloidogenicity. High contiguity of the final assemblies allowed us to trace back the history of reciprocal unbalanced translocations between chromosomes I, VIII, IX, XI, and XVI of the PGC strains. We show that formation of hybrid alleles of the *FLO* genes during such chromosomal rearrangements is likely responsible for the lack of invasive growth of yeast strains. Taken together, our results highlight important features of laboratory yeast strain evolution using the power of long-read sequencing.

## Introduction 

Third generation sequencing methods are becoming a new gold standard in the field of genomics. Unlike second generation short-read technologies, third generation sequencing allows one to obtain long (up to 2,000,000 nucleotides) sequencing reads which are of ultimate importance for repeat resolution and scaffolding (for review, see (Goodwin *et al.* 2016)). Both of the widely used third generation sequencing platforms, Pacific Biosciences (PacBio) and Oxford Nanopore Technologies (ONT), are increasingly used for genomic analysis of a wide range of species, including model organisms.

One of the most extensively studied model organisms, baker’s yeast *Saccharomyces cerevisiae*, is a widely used system for studying molecular and cell biology, genetics, and evolution. Genomes of numerous natural isolates and laboratory strains of *S. cerevisiae* have been sequenced with both short and long read technologies (*e.g.*, Istace *et al.* 2017; Peter *et al.* 2018). One of the recent studies has revealed important trends in the genome evolution of laboratory yeast strains, namely: (i) fast accumulation of unbalanced structural rearrangements as opposed to balanced ones; (ii) rapid reshuffling of ectopic chromosomal sequences; and (iii) faster molecular evolution in subtelomeric regions (Yue *et al.* 2017). A later effort to sequence a large compendium of more than 1,000 yeast strains of diverse origin has also provided important insights into the prevalence of different types of structural variants (SVs) in wild *S. cerevisiae* strains, including widespread loss of heterozygosity and a critical role of copy number variants (CNVs) in phenotypic diversity (Peter *et al.* 2018).

Peterhof Genetic Collection (PGC) is a large laboratory stock of *S. cerevisiae* strains that have been isolated independently from the reference S288C lineage, though PGC strains have been crossed with S288C lineage strains several times during their evolution (Drozdova *et al.* 2016b). Whole-genome sequencing of several major PGC strains using short read technologies allowed to generate novel insights into the diversity of PGC genomes, including the analysis of aneuploidies, as well as identification of functionally relevant mutations in flocculation-related genes (Drozdova *et al.* 2016b). At the same time, no information about SVs and chromosomal rearrangements in PGC strains has been obtained to date. As PGC stock is (at least in part) independent from major laboratory strains, it can be used as an important source to validate key assumptions about laboratory strain evolution. To address this question, we performed Oxford Nanopore sequencing of two widely used strains from the PGC collection: U-1A-D1628 and 74-D694.

The 74-D694 and U-1A-D1628 strains were chosen as the most frequently used among PGC for the research in our and many other laboratories. These two strains are closely related, but are not isogenic and differ in auxotrophies. The 74-D694 strain, its derivatives OT55 and OT56, as well as GT81 (isogenic to U-1A-D1628) are often used to study yeast prions and amyloids (Chernoff *et al.* 1995; Newnam *et al.* 1999; Wang *et al.* 2017). Bearing a deletion of the chromosomal *SUP45* (eRF1-encoding) gene and nonsense mutations of different types, 1A-D1628 was very helpful for studies of termination of translation (Le Goff *et al.* 2002; Moskalenko *et al.* 2003; Petrova *et al.* 2015), and is still widely used in this field.

## Materials and methods

### Yeast strains and media

Standard methods of cultivation and manipulation of yeast and bacteria were used throughout this work (Kaiser *et al.* 1994; Sambrook *et al.* 1989). *S. cerevisiae* strains used in this work are listed in Table 1. U-1A-D1628 is a 1A-D1628 derivative containing pRS316-SUP45 plasmid with a *URA3* selectable marker (Moskalenko *et al.* 2003) as a source of the essential *SUP45* gene. S45-1A-D1628 is a U-1A-D1628 derivative in which the *SUP45* gene is returned back to its native chromosomal location. It was obtained in this work by transforming U-1A-D1628 with linearized pRS316-SUP45 vector and subsequent selection of the transformants on 5-FOA medium for counter-selection of the *URA3*-containing plasmids (Kaiser *et al.* 1994).

**Table 1.** Yeast strains used in this work

Strain	Genotype	Reference	Source
**U-1A-D1628**	*MATα ade1-14(UGA) trp1-289(UAG) his3 lys2 ura3-52 leu2-3,112 sup45::HIS3MX* [pRS316-SUP45] [*psi*^-^] [*PIN*^+^]	(Le Goff *et al.* 2002)	Laboratory collection
**74-D694**	*MATa ade1-14(UGA) trp1-289(UAG) ura3-52 his3-Δ200 leu2-3,112 flo8-1* [*psi*^-^] [*PIN*^+^]	(Chernoff *et al.* 1995)	Laboratory collection
**10560-23C**	Sigma1278b background: *MATα ura3-52 his3::hisG leu2::hisG*	(Robertson and Fink 1998)	Gift from H.-U. Mösch
**10560-6B**	Sigma1278b background: *MATα ura3-52 his3::hisG leu2::hisG trp1::hisG*	(Lo *et al.* 1997)	Gift from H.-U. Mösch
**BY4742**	*MATα his3Δ1 leu2Δ0 lys2Δ0 ura3Δ0*	(Brachmann *et al.* 1998)	Gift from Y.I. Pavlov
**S1 (S288C)**	*MATα SUC2 gal2 mal2 mel flo1 flo8-1 hap1 ho bio1 bio6*	(Mortimer and Johnston 1986; Strope *et al.* 2015)	Gift from J. McCusker
**W1588-4C (W303-1A)**	*MATa ade2-1 can1-100 his3-11,15 leu2-3,112 trp1-1 ura3-1 RAD5*	(Thomas and Rothstein 1989; McDonald *et al.* 1997)	Gift from T. Petes
**S45-1A-D1628**	*MATα ade1-14(UGA) trp1-289(UAG) his3 lys2 ura3-52 leu2-3,112 SUP45* [*psi*^-^] [*PIN*^+^]	This work	Laboratory collection

### Genomic DNA extraction

For long genomic DNA sequencing, yeast strains were grown in 250 mL of YPD medium (1% yeast extract, 2% pepton, and 2% glucose) with additional adenine (final concentration 40 mg/L) until OD_600_ of 2.0. Cells were collected by centrifugation in a 50 mL falcon tube for 10 minutes at 4,500 rpm, washed with 50 mL of distilled water, and precipitated again. Precipitated cells were resuspended in 10 mL of lysis buffer (1 M sorbitol, 0.05 M EDTA, pH 8.0) and treated with 0.1 mL of zymolyase 20 T solution (12 mg/mL) for 1 hour at 37^∘^C. Resulting spheroplasts were spun down by centrifugation (10  minutes at 4,500 rpm). The pellet was resuspended in 10 mL of TLB buffer (0.1 M NaCL, 0.01 M Tris-HCl, 0.025 M EDTA, 0.5% (w/v) SDS, 20 μg/ml of RNAse A added right before use). The solution was incubated for 2 hours at 37^∘^C. The solution was then treated by proteinase K (Thermo Scientific), added to a final concentration of 200 μg/mL, and incubated for 5 hours at 50^∘^C. After this, 10 mL of UltraPure^TM^ Buffer-Saturated Phenol (Thermo Scientific) was added. The mixture was incubated for 10 minutes at room temperature with gentle rotation and centrifuged for 10 minutes at 4,500 rpm. Phases were separated and the upper phase was treated with 5 mL UltraPure^TM^ Buffer-Saturated Phenol (Thermo Scientific) and 5 mL of 24:1 chloroform—isoamyl alcohol mixture. The mixture was again centrifuged for 10 minutes at 4,500 rpm and upper phase was collected.

The solution was then treated with 4 mL of 5 M ammonium acetate and 30 mL of ice cold 98% ethanol. This resulted in DNA precipitation in the form of large jellyfish-like clump, which was transferred into a clean tube using a glass hook. The DNA was washed first with 1 mL of 70% ethanol, and then with 1 mL of 98% ethanol, precipitated by quickly spinning it at 10,000 rpm and dried at 55^°^C for 5 minutes. The resulting precipitate was incubated overnight at 4^∘^C in 0.1 mL of elution buffer (0.01 M Tris-HCl, 0.05% Triton-X100, pH 8.0) to allow its complete dissolution. Concentration of DNA was determined to be approximately 3 mg/mL. The solution was diluted tenfold before the library preparation.

For all purposes other than Nanopore sequencing, genomic DNA was extracted using the same protocol with the following modifications: first, the solution was not treated by proteinase K; second, precipitated DNA was dissolved in 0.1 mL of deionized water. The concentration and quality of genomic DNA (gDNA) were monitored by agarose gel electrophoresis and NanoDrop spectrophotometer (Thermo Scientific).

### Genome sequencing

Sequencing of the two yeast genomes was performed using Oxford Nanopore MinION sequencer with 9.4.1 flowcells. Sequencing using modified protocol (https://www.protocols.io/view/ultra-long-read-sequencing-protocol-for-rad004-mrxc57n) based on rapid protocol (SQK-RAD002) to obtain ultra-long DNA reads was attempted and failed, presumably due to pore contamination with a fungal metabolite or other impurities. Sequencing using native barcode ligation protocol (SQK-LSK109) was successful, generating 10 Gb (U-1A-D1628 strain) and 7.5 Gb (74-D694 strain) of raw sequence, respectively. Using estimated genome size of 12.2 Mb, this corresponds to ≈ 800x coverage for the U-1A-D1628 strain and ≈ 600x coverage for the 74-D694 strain.

For short read Illumina sequencing, we fragmented 500 ng of DNA using NEBNext^®^ dsDNA Fragmentase^®^ (NEB, USA), following the manufacturer’s recommendations. We then purified the fragmented DNA using Ampure XP beads (Beckman, USA) and prepared DNA libraries using the Kapa HTP library preparation kit (Roche, Switzerland) according to the original protocol. We used 6 cycles of PCR for the final library amplification and conducted dual size selection after the final PCR aiming at 550 bp library size. We sequenced the yeast DNA library on Illumina HiSeq 2500 platform in a paired-end mode with the read length of either 2 × 130 or 2 × 150 bp.

### Genome assembly

Resulting fast5 files were basecalled using high-accuracy mode of guppy v. 3.1.5, and assembled with canu v. 1.8 (Koren *et al.* 2017) with default Oxford Nanopore raw read presets. Given the excessive coverage, we also used filtlong v. 0.2.0 (https://github.com/rrwick/Filtlong) to obtain a 100x subset of Nanopore reads with best quality and length. The resulting assemblies were polished with signal-level raw data using Nanopolish v. 0.9.2 (Loman *et al.* 2015), after which Illumina-based polishing using Racon v. 1.4.10 (Vaser et al. 2017) was used. All resulting assemblies were evaluated using QUAST v. 5.0.2 (Gurevich *et al.* 2013) and BUSCO v. 4.1.2 (Waterhouse *et al.*2018) using “saccharomycetes_odb10” reference database, and “saccharomyces_cerevisiae_S288C” species parameter for Augustus gene finding.

For hybrid assembly of circular sequences (*i.e.*, mitochondrial DNA and plasmids) with both ONT and Illumina reads we used Unicycler v. 0.4.9b (Wick *et al.*2017) with recommended settings. The resulting contigs were aligned against the S288C mitochondrion (RefSeq ID: NC_001224.1), 2-micron DNA (RefSeq ID: NC_001398.1), or the pRS316-SUP45 plasmid to select matching sequences. Detailed comparisons were made by constructing a multiple sequence alignment using MAFFT v. 7.310 (Katoh and Standley 2013) or ClustalW v. 1.81 (Thompson *et al.*1994) and visualized using a custom set of scripts. Circular mtDNA and 2-micron DNA sequences were rotated using a custom script [available in the project repository (see Data Availability)] to start at the same location.

### Transposable element location analysis and tree construction

Yeast transposable element (Ty) sequences of the S288C strain were downloaded from the *Saccharomyces* Genome Database (http://yeastgenome.org). These sequences were uniformly mapped to all assemblies under consideration using NCBI BLAST+ v. 2.6.0 (Altschul *et al.* 1990), and only locations with at least 95% sequence identity and at least 95% coverage of a complete Ty sequence were retained. To analyze the number of shared Ty element locations, we extracted the sequence of each Ty element from the genome using bedtools (Quinlan and Hall 2010), adding 1,500 nucleotides of upstream and downstream genomic sequence. The resulting sequences were compared using blastn, and the location was considered to be conserved between strains if the complete sequence were matched with at least 95% identity and 99% of query sequence coverage. To construct the neighbor joining tree, the proportion of shared and distinct Ty element locations was calculated, and the latter number was used as a distance measure for the analysis. The tree was constructed and visualized using the ape R package (Paradis and Schliep 2019). To compare the resulting tree with the one derived from SNP data, genome assemblies were aligned to each other using minimap2, and the number of pairwise substitutions was calculated using paftools (https://github.com/lh3/minimap2/tree/master/misc).

### Structural variant analysis

For SV analysis, we used the subsets of 100x ONT reads with the highest quality selected using filtlong v. 0.2.0. These read subsets for each strain were aligned onto the reference genome of the S288C strain using NGMLR and SV calling was performed using the Sniffles package (Sedlazeck *et al.* 2018) with the default settings. Sets of called SVs were manually curated to filter out false positive calls without sufficient support.

For the analysis of copy-number variants, we also employed the samplot package (https://github.com/ryanlayer/samplot) to visualize read coverage profiles in the regions of interest. We used Nanopore reads of S288C from (Giordano *et al.* 2017) (NCBI Sequencing Read Archive (SRA) accessions ERR1883402, ERR1883401, ERR1883400, ERR1883399, ERR1883398, and ERR1883389). An alternative assembly of S288C (Genbank accession No GCA_002057635.1), constructed from long reads (PacBio) (Yue *et al.* 2017), was also used. Numerical estimates of short read coverage were made using bedtools (Quinlan and Hall 2010).

For visualization of chromosomal translocations we aligned genome assemblies of U-1A-D1628 and 74-D694 strains to the reference genome of S288C with MashMap v. 2.0 (Jain *et al.* 2018) using threshold for identity as 96% and mapping segment length longer than 5,000 bp. Alignments were depicted as circos plots using Circa v. 1.2.0 (http://omgenomics.com/circa)

### Amyloidogenicity prediction

For the analysis of the amyloidogenic potential of protein variants, *ab initio* gene prediction was performed for each genome assembly using Augustus (Stanke and Morgenstern 2005) software with the “saccharomyces_cerevisiae_S288C” species parameter. Predicted coding sequences were compared against the yeast reference proteome using BLAST, and records matching reference proteins of interest were selected. Amyloidogenicity was predicted using ArchCandy v. 1.0. (Ahmed *et al.* 2015). Cumulative arch score was used as a quantitative measure of amyloidogenic potential and calculated as the sum of all scores of the beta arches, which include this amino acid. The MUSCLE algorithm was used to construct the alignment of protein sequences (Edgar 2004). The gaps in the alignment were considered to have 0 arch scores when creating the plot with cumulative arch score.

### qPCR

The copy number of target genome fragments was analyzed by quantitative PCR (qPCR) with EVA Green 2.5x PCR-mix (Syntol) according to the manufacturer instructions. Primer pairs used in this analysis are listed in Supplementary Table S1. Reactions and quantification were performed using CFX96 amplifier (Bio-Rad, Hercules). The quantitation cycle for *ACT1* was used as a reference. Triplicate qPCRs were performed for each biological replicate. The ΔΔC_*T*_ method (Livak and Schmittgen 2001) was used to measure the relative fold quantification. Resulting values were used to estimate the number of *CUP1* cluster copies.

### PCR-based SV validation

For PCR-based validation of SVs, we amplified the corresponding genomic regions using custom primers (see Supplementary Table S1). For each reaction, 500 ng of gDNA was used. Amplification was performed with BioRad T100 Thermal Cycler. PCR products were analyzed using both gel electrophoresis and Sanger sequencing. For sequencing, DNA fragments were isolated using GeneJET PCR Purification Kit (Thermo Scientific) and sequenced at the RRC MCT SPbU.

### Invasive growth assay

Invasive growth assay was performed as previously described (Roberts and Fink 1994). Transformants were plated onto YPD plates with a toothpick. Prior to invasive growth analysis, plates were incubated for 5 days at 30^∘^C and then for 2 days at room temperature. Nonadhesive cells were washed off the agar with a gentle stream of water, leaving subsurface cells that had invaded the agar. Plates were photographed following the wash. All analyses were performed in triplicate. Plasmids harboring reference *FLO* genes were extracted from the following clones from the YSC4613 Yeast genomic tiling collection (Jones *et al.* 2008): YGPM33d10 (*FLO1*), YGPM32m17 (*FLO5*), YGPM14m17 (*FLO8*), and YGPM13d18 (*FLO11*).

### Comparative genomic hybridization (CGH)

Extraction and labeling of genomic DNA from the U-1A-D1628 strain for the microarray-based CGH was performed as described (Zhang *et al.*2013). Custom 8x15k design (AMID 028943) Agilent arrays were used. CGH analysis was carried out as described previously (Drozdova *et al.* 2016a). The data were processed with the CGH-Miner Excel add-in (Wang et al., 2005) and a custom R function based on the clac package.

## Data availability

All data pertinent to this project are deposited at NCBI under BioProject number PRJNA656310 (BioSample IDs SAMN15777460 and SAMN15777459). Sequencing reads have been uploaded to the NCBI SRA under accessions SRR12427676 and SRR12427674 (Nanopore sequencing data), and SRR12427673 and SRR12427675 (Illumina sequencing data). Resulting genome assemblies have been deposited to NCBI Genome under accessions GCA_014898825.1 (U-1A-D1628), and GCA_014898935.1 (74-D694). Additional data files, scripts, and final assemblies are available at GitHub: https://github.com/mrbarbitoff/pgc_genomes_ont. Supplemental Material is available at figshare: https://doi.org/10.25387/g3.13635110.

## Results and discussion

### Construction and primary analysis of the genome assemblies

**Figure 1:**
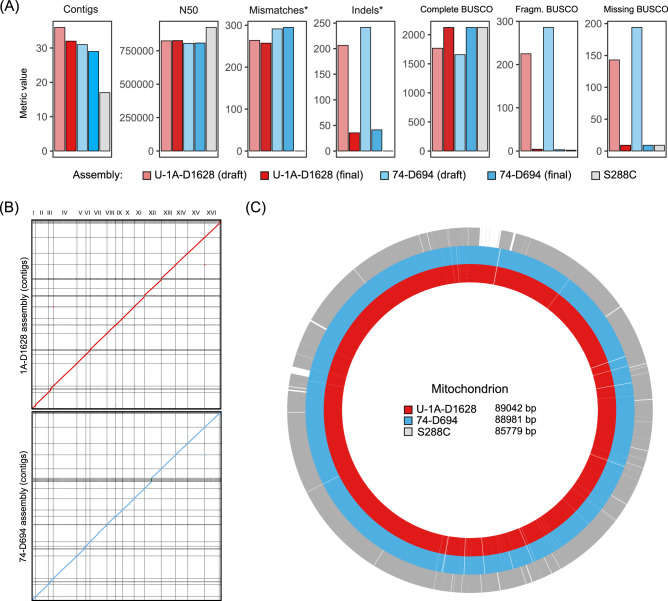
Chromosome-level assembly of the PGC strain genomes. (A) Major quality statistics for the final assemblies of U-1A-D1628 and 74-D694 genomes compared to the S288C reference. Assembly metrics were estimated using QUAST (Gurevich *et al.* 2013). * indicates that number of SNPs and indels per 100 kbp were estimated using S288C sequence as reference. Number of BUSCOs was estimated using saccharomycetes_odb10 lineage-specific database. (B) Dotplot visualization of the whole-genome alignment of U-1A-D1628 and 74-D694 assemblies onto the S288C reference genome. Plots were obtained using D-GENIES (http://dgenies.toulouse.inra.fr). (C) Alignment of the mitochondrial genome sequences of the respective strains. For PGC strains, mitochondrial genome sequence was assembled using a hybrid assembly framework. Circular mtDNA sequences were aligned using MAFFT and the resulting MSA was visualized using a custom set of scripts. The resulting picture was manually corrected to reflect alignment gaps in repetitive regions.

We used ONT MinION sequencing to construct chromosome-scale genome assemblies for our strains. We sequenced both U-1A-D1628 and 74-D694 genomes with the 1 D sequencing protocol, which yielded 10.0 and 7.5 Gbp of sequencing data for U-1A-D1628 and 74-D694 strain, respectively. Mean read lengths were comparable for both strains (7.4 and 6.0 kbp, respectively). Draft genome assemblies showed a high degree of contiguity with only slightly lower N50 value compared to the S288C reference (Table 2; Figure 1A) but relatively low base accuracy, as expected. After constructing the draft assemblies we then conducted a two-step polishing of the assembly using both raw ONT signal with Nanopolish and Illumina sequencing reads for these strains. Final polished assemblies showed high contiguity and base accuracy (Figure 1A). We also assessed the number of benchmarking universal single-copy orthologs (BUSCOs) (Waterhouse *et al.* 2018) of the *Saccharomycetes* class that can be found in the resulting final assemblies. We found that the assembled genomes of our strains had almost exactly the same numbers of complete, fragmented, and missing BUSCOs when compared with the S288C reference genome (Table 2, Figure 1A), with a difference in 1 or 2 fragmented genes being attributable to the genotypes of the strains. Taken together, these results suggest that our assemblies have very high contiguity and base accuracy and are similar or better in quality compared to the other long read-based genome assemblies (*e.g.*, (Istace *et al.*2017; Matheson *et al.* 2017)).

**Table 2.** Comparison of the main assembly statistics for the sequenced genomes

Quality metric	U-1A-D1628 (draft)	**U-1A-D1628 (final)** ^a^	74-D694 (draft)	**74-D694 (final)** ^a^	S288C
**Number of contigs**	36	32	31	29	17
**N50 (kbp)**	824	825	806	808	924
**Mismatches per 100 kbp**	263.9	257.3	291.3	294.8	n.a.
**Indels per 100 kbp**	206.5	35.4	241.5	41.4	n.a.
**Complete BUSCOs**	1,769	2,124	1,657	2,125	2,126
**Fragmented BUSCOs**	225	4	286	3	2
**Missing BUSCOs**	143	9	194	9	9

a Contigs corresponding to the mitochondrion sequences were replaced by a complete circular mitochondrial DNA sequence obtained by hybrid assembly prior to quality control for both final assemblies.

We then conducted whole-genome alignment to assess the structural similarity of the PGC strain genomes with the S288C. We found that both U-1A-D1628 and 74-D694 assemblies showed high degree of similarity to the S288C reference (Figure 1B), with no large-scale chromosomal abnormalities. Importantly, all but two (in case of U-1A-D1628) or three (in case of 74-D694) chromosomes were assembled into a single contig. Neither mitochondrial genome nor any of the plasmids, however, were assembled into a single contig. To construct a single circular mitochondrial genome sequence, as well as to investigate whether a complete 2-micron DNA is present in our strains, we applied a hybrid assembly approach with Unicycler (Wick *et al.* 2017) using both ONT and Illumina data at once. Hybrid assembly approach allowed us to reconstruct the complete mitochondrial genome of both U-1A-D1628 and 74-D694, with both sequences being highly similar to one another (Figure 1C). Interestingly, we found that both assembled mitochondrial genomes were around 3 kbp longer compared to the reference mitochondrial genome sequence of the S288C strain. The size of the mitochondrial genome is known to significantly vary in yeast (De Chiara *et al.* 2020), with many yeast strains having mitochondrial genomes of > 90,000 bp. Hence, it may be hypothesized that the mitochondrial genome of the PGC strains originates from an ancestral lineage independent from the S288C one, and that the increased size of the mitochondrial genome was characteristic of this ancestral lineage. Several additional sequence insertions in repetitive A/T-rich DNA regions are found in PGC mitochondrial DNA compared to S288C, with the largest (1.7 kbp long) insertion located downstream of the *ATF6* gene. Repetitive intergenic sequences can be considered as a driver of structural variation in mitochondrial genomes (De Chiara *et al.* 2020); and the larger insertions could have resulted from erroneous recombination, which always occurs in yeast mitochondria (Dujon 2020).

Two of the smaller insertions corresponded to the coding sequence of the intron-encoded DNA endonuclease aI5 beta, resulting in-frame insertions of 11 and 13 amino acids, respectively. The presence of additional asparagines in the mitochondrial ribosomal protein Var1 (uS3m) was also noted. Such variation in the *VAR1* sequence was described before (Hudspeth *et al.* 1984). Yeast Var1 protein is rich in asparagine residues (near 30% of all content) which are distributed on the protein surface (Desai *et al.* 2017), however, the biological sense of these asparagine variations is unknown. Other amino acid substitutions in mitochondrial proteins of sequenced strains are listed in Supplementary Table S2. Similarly to what has previously been reported for laboratory yeast strains (Yue *et al.* 2017), no other structural rearrangements in the mitochondrial genome were observed, and the sequence collinearity was maintained when compared to S288C. Detailed analysis of hybrid assembly results also showed that both U-1A-D1628 and 74-D694 strains harbor a full-length 2-micron DNA sequence. 2-micron DNA is known to exist in two forms (A and B), which differ in one large inversion (Broach 1982). Interestingly, A-form of 2-micron DNA was assembled in 74-D694 while B-form was found in U-1A-D1628. We constructed an A-form for U-1A-D1628 and B-form for the 74-D694 and compared them to the reference 2-micron sequences. In both cases the 2-micron sequences were nearly identical to the reference one, with one single-nucleotide substitution and a 125 bp deletion (Supplementary Figure S1).

### Transposable element location in PGC strain genomes

**Figure 2:**
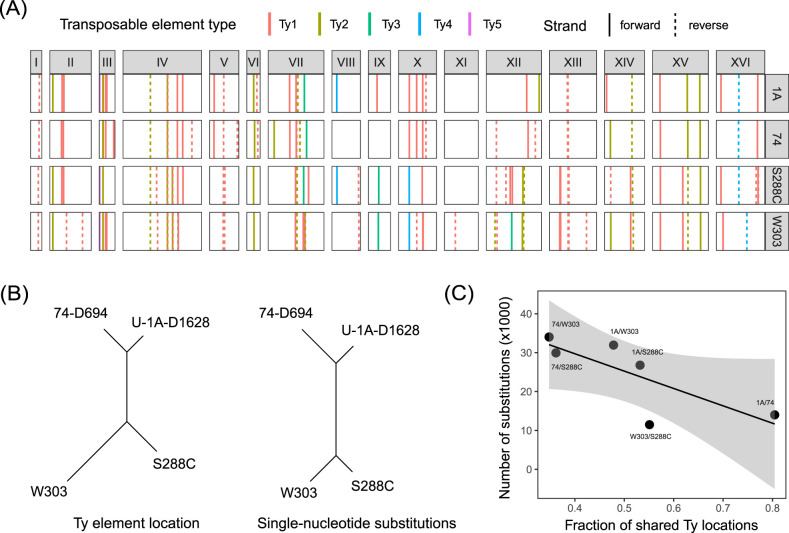
Analysis of TE location in the genomes of U-1A-D1628, 74-D694, S288C, and W303 strains. (A) Locations of complete Ty elements in the genomes of the indicated strains. For U-1A-D1628 (designated as “1A”), 74-D694 (“74”), and W303 short contigs were omitted. Line type indicates whether an element matches the contig in forward or reverse direction. (B) NJ trees constructed using the proportion of shared Ty element locations (left) or the number of single-nucleotide substitutions (right) estimated by assembly-to-reference alignment of complete genomes. (C). A scatterplot showing the relationship between pairwise fraction of shared Ty element locations and the number of single-nucleotide substitutions.

We then turned our attention to the analysis of transposable elements (TEs) and their genomic location. Third-generation sequencing and complete genome assemblies allow to accurately locate all TEs in the genome, and evaluate both total content of TEs in a genome and their mobility in the recent history of a species (Istace *et al.* 2017). To map TE sequences in the genomes of our strains, we used reference sequences of Ty elements from the S288C genome (see Materials and Methods). We detected a total of 47 and 46 complete Ty elements in the genomes of U-1A-D1628 and 74-D694, respectively. Of these, 42 and 43 elements, respectively, were located in large chromosome-scale contigs. To analyze the evolutionary conservation of the Ty element location, we first visually compared the distribution of the Ty sequences across yeast chromosomes for our strains and two reference strains, the S288C and its relative, W303 strain, which has also been sequenced recently using third-generation technologies (Matheson *et al.* 2017). We found that the location of TEs was highly similar for the U-1A-D1628 and 74-D694 (Figure 2A), which shared 37 TE locations. As expected, PGC strains shared fewer TE locations with the S288C (25 and 22 for U-1A-D1628 and 74-D694, respectively) and even less with W303 (17 and 16, respectively). The orientation of Ty elements at each location was nearly identical across strains, with the exception of one element on chromosome XV (Figure 2A). Given these results, we next hypothesized that the fraction of shared TE locations is proportional to the evolutionary distance between strains. To evaluate this hypothesis, we constructed a neighbor-joining tree using the fraction of distinct Ty elements as the distance measured and compared the topology of this tree with the one constructed using the number of single-nucleotidesubstitutions. Indeed, we found that the trees were highly similar to each other (Figure 2B), though the divergence between W303 and S288C was overestimated when using Ty data for tree construction. Consistent with these findings, we observed a good correlation between the number of SNPs and the fraction of shared Ty element locations (Figure 2C).

### Overview of the structural rearrangements in PGC genomes

We next turned to explore the landscape of SVs in the U-1A-D1628 and 74-D694 genomes. To do so, we aligned the raw ONT reads against the S288C reference genome using NGMLR (Sedlazeck *et al.* 2018) and then called SVs using Sniffles (Supplementary Table S3). The resulting SV calls were then manually curated, specifically focusing on variants spanning protein-coding genes. We discovered 49 and 55 SVs overlapping with the protein-coding regions (excluding transposons) of 42 and 45 genes in the U-1A-D1628 and 74-D694, respectively (Supplementary Table S4).

**Figure 3:**
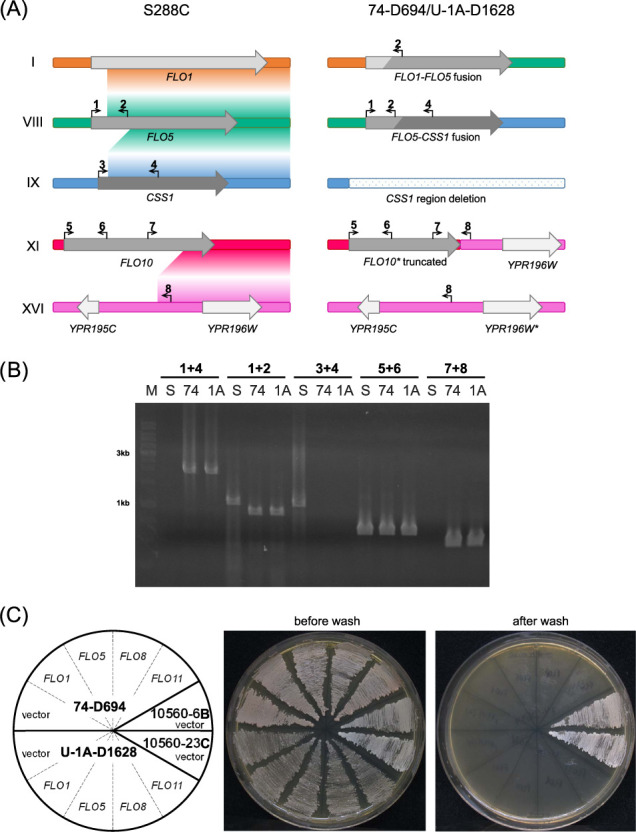
Translocations detected in the strains and their influence on the invasive growth. (A) Translocations in 74-D694 and U-1A-D1628 strains affecting *FLO* genes. Gradient-colored fields between reference chromosomes depict possible translocation routes. Genes, nonfunctional due to frameshift mutations, are marked with an asterisk. (B) Confirmation of detected translocations with PCR. Numbers correspond to primers designated on (A); S, 74, and 1A correspond to S1, 74-D694, and U-1A-D1628 genomic DNA, used as a template; M, DNA molecular weight marker (SibEnzyme, 1 kb). (C). Agar invasion visualized using the wash test. The *S. cerevisiae* strains U-1A-D1628 and 74-D694, were transformed with plasmids containing *FLO1, FLO5, FLO8*, or *FLO11* genes and streaked onto YPD plates. Plates were photographed before and after invasive growth assay (see Materials and Methods). As a positive control invasive growth was assessed for 10560-6B and 10560-23C yeast strains.

We first noticed that some of the SVs identified affected the sequences of the *FLO* genes that play an important role in establishment of the cell-to-cell contacts, flocculation, and invasive growth. Structural rearrangements and reshuffling of ectopic chromosome sequences has been shown to affect the number of functional *FLO* genes. Importantly, it is hard to reconstruct the exact type and sequence of chromosomal rearrangements in subtelomeric regions due to high similarity of these sequences. Such a similarity hinders accurate one-to-one matching of chromosome ends when conducting whole-genome alignment (Supplementary Figure S2). Given this complication, we curated candidate translocations involving telomeric and subtelomeric sequences by manual inspection of pairwise sequence alignment. Such an inspection allowed us to trace the history of rearrangements between several chromosomes (Figure 3, A and B). Our analysis showed that the region corresponding to the chromosome I was substituted with a chromosome VIII subtelomeric region as the result of recombination between the *FLO1* and *FLO5* paralogs (Figure 3A). The remaining 5’-end of the *FLO5* gene fused with the 3’-end of the *CSS1* gene, located on chromosome IX in reference strain. The remaining part of the *CSS1* region of chromosome IX is absent in the PGC strains, which is confirmed in U-1A-D1628 by CGH array (Supplementary Figure S3, Supplementary Table S5). Finally, we detected a translocation of the duplicated chromosome XVI right subtelomeric region in place of the chromosome XI fragment starting from the 3’-part of the *FLO10* gene. This translocation is seen as loss of chromosome XI fragment and duplication of chromosome XVI region on the CGH array plot (Supplementary Figure S3, Supplementary Table S5). The truncated *FLO10* gene is additionally inactivated by the frameshift mutation (Figure 3A), which was confirmed by Sanger sequencing of the region.

Previous studies have shown that, despite no obvious difference in cellular aggregation of U-1A-D1628 and 74-D694 was observed, the PGC strains differ in the *FLO8* alleles, which is functional in the 1A-D1628, but, similar to the reference strain, contains a premature stop codon in 74-D694 (Petrova *et al.* 2015; Drozdova *et al.* 2016b). However, both strains contain missense mutation in *AMN1*, also known to contribute to the cell clumping phenotype (Li *et al.* 2013). We then questioned whether extensive exchanges of the telomere and subtelomere sequences also affect the functionality of the *FLO* genes located in these regions. One can suggest two alternative models of the evolution of these sequences: (i) PGC strains, derived from an independent industrial lineage, preserved the ancestral sequence of the subtelomeric regions and all affected genes retained their functional ancestral sequence; (ii) PGC strains suffered additional structural rearrangements during their laboratory evolution, resulting in disruption of the remaining functional genes of the *FLO* family (*FLO1, FLO5*, and *FLO8*).

To shed light on the functionality of the flocculin genes, we conducted an invasive growth assay comparing PGC strains with and without additional expression of the reference *FLO* genes. To this end, PGC strains were transformed with the plasmids derived from the YSC4613 Yeast genomic tiling collection (Jones *et al.* 2008) harboring different *FLO* genes (*FLO1, FLO5, FLO8*, and *FLO11*). We used two derivatives of the Sigma1278b strain as the reference for comparison. Our analysis showed that neither U-1A-D1628 nor 74-D694 could efficiently invade into agar even upon additional expression of the *FLO* genes (Figure 3C). In some replicates, expression of the reference *FLO1* gene was partially restoring the phenotype; however, this result was not reproducible across biological replicates. It seems that too many genes involved in yeast multicellularity are affected both by structural rearrangements and intragenic variations, so that “repairing” only one component of the system does not rescue its functionality. Hence, we can conclude that additional structural aberrations in the subtelomeric sequences resulted in nearly complete deactivation of the *FLO* genes in PGC strains.

### Copy number variation in PGC strain genomes

There are several regions of the yeast core genome with known natural variation in the copy number. These regions include the copper metabolism *CUP1* gene cluster and the *ENA* sodium ATPase pumps (Istace *et al.* 2017).

**Figure 4:**
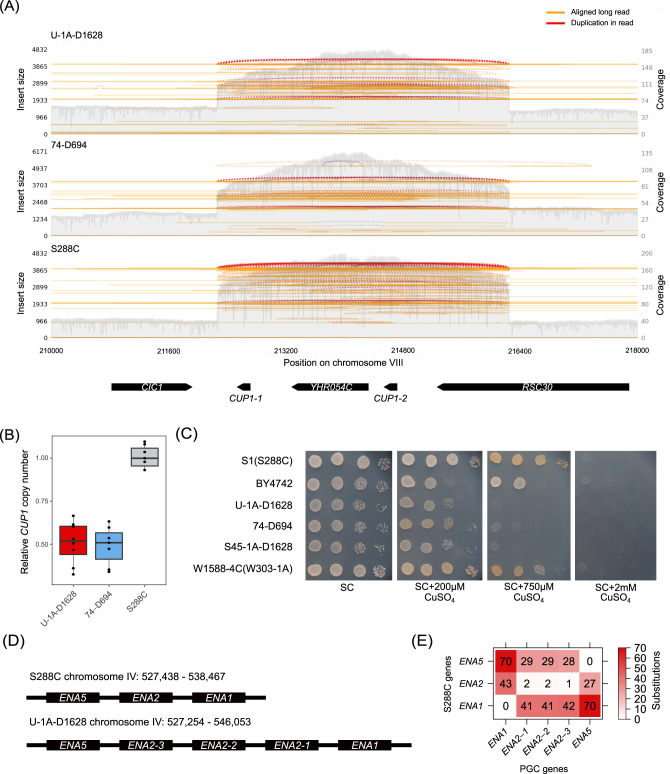
Analysis of *CUP1* and *ENA* gene copy number. (A) Visualization of the long read coverage profiles in the region of chromosome VIII spanning the *CUP1* cluster. Orange lines represent aligned long reads, red dashed lines represent reads supporting duplication of the region. S288C reads are taken from the study by Giordano *et al.* (2017) (see Materials and Methods). (B) Number of *CUP1* gene copies relative to S288C estimated using qPCR analysis. Each point represents an independent biological replicate. (C) Copper sensitivity of different strains assessed by growth assay. Cells of the respective strains were plated onto the SC media containing indicated concentrations of CuSO_4_. Tenfold serial dilutions are shown. (D) Schematic representation of the genes in the *ENA* locus on chromosome IV for S288C (top) and PGC strains (bottom). For the 74-D694 strain, the *ENA* locus is identical to U-1A-D1628 and is located at chromosome IV: 527,320–546,117. (E) A heatmap representation of the numbers of single-nucleotide substitutions between the coding sequences of the *ENA* genes from the PGC strains and S288C.

We first evaluated the copy number of the *CUP1* gene cluster that is known to naturally vary across different strains of *S. cerevisiae*. Visual inspection of whole genome alignment and the distribution of mapped reads suggested that the PGC strains harbor at least three times more copies of the corresponding region on chromosome VIII of the reference S288C genome (Figure 4A). Concordantly with these observations, automated detection of the *CUP1* gene promoters in the assembled genome sequences showed that both U-1A-D1628 and 74-D694 genomes contained 8 copies of the *CUP1* gene, in contrast to only two such copies in the reference genome of S288C. Since known estimates of the copy number of *CUP1* locus in S288C greatly exceed the two copies present in reference genome assembly (Zhao *et al.*2014), we did the same mapping with Oxford Nanopore reads of S288C (Giordano *et al.* 2017). Indeed, the relative coverage of the region appeared approximately fivefold, which is even higher than in the PGC strains (Figure 4A).

We then used qPCR to validate the differences in the *CUP1* copy number. Indeed, we found that the relative number of the *CUP1* gene copies estimated by qPCR was approximately twofold lower for the PGC strains compared to the reference one (Figure 4B) which corroborates long read alignment data. In addition, we conducted a phenotypic assay to evaluate the resistance of the strains to varying concentrations of copper sulfate in the medium. As histidine auxotrophy is known to affect copper resistance in yeast (Pearce and Sherman 1999), we added His^-^ BY4742 (isogenic to S288C) and S45-1A-D1628 (isogenic to U-1A-D1628) strains to the analysis. The results of this analysis confirmed the qPCR results, as both U-1A-D1628 and 74-D694 cells showed lower viability in the presence of high CuSO_4_ concentrations (Figure 4C) compared to S288C (S1) and related laboratory strains, such as W303 and a commonly used BY4742 strain. The high sensitivity of U-1A-D1628 strain to high concentration of copper could not be explained by the presence of a plasmid-borne copy of *SUP45*, as the S45-1A-D1628 strain with the full-length *SUP45* gene integrated back into the chromosome also had low viability when exposed to high concentrations of copper. Taken together, these results indicate that the S288C genome harbors larger number of *CUP1* copies compared to the reference assembly. The assemblies of U-1A-D1628 and 74-D694 were both shown to contain 8 copies of the *CUP1* locus, therefore, based on the qPCR data, the S1 (S288C) strain should contain twice as much. This assumption is corroborated by earlier analysis conducted with Southern blot hybridization (Zhao *et al.*2014). Data on the *CUP1* copy number estimation in reference and PGC strains are summarized in Supplementary Table S6.

We also analyzed the copy number variation at the *ENA* locus. The number of *ENA* gene copies has been shown to greatly vary across strains of *S. cerevisiae* with up to 14 copies per genome (Strope *et al.* 2015). Coverage profile of both ONT and Illumina reads suggested a possible duplication of the *ENA1* and *ENA2* genes; however, coverage was very nonuniform, and reads with ambiguous alignment coordinates hindered making a confident conclusion (Supplementary Figure S4). A more detailed BLAST-based analysis of the corresponding region in the PGC genome assemblies allowed us to identify three copies of the *ENA2* gene flanked by one copy of *ENA1* and *ENA5* (Figure 4D). While the *ENA1* and *ENA5* coding sequences of the PGC strains were absolutely identical to the S288C genome, each copy of the *ENA2* harbored one or two substitutions with respect to the reference *ENA2* coding sequence (Figure 4E). The results of the sequence comparison suggest that the *ENA2-3* gene is the likely ancestral copy, while *ENA2-1* and *ENA2-2* arose through a second gene duplication event and share an additional mutation.

### Tandem repeat variation in protein-coding genes

Out of the grand total of 62 curated SVs in the U-1A-D1628 and 74-D694 genomes, a significant proportion were located in repeat sequences, with many of these encoding potentially amyloidogenic proteins. This result is especially interesting given that the studied yeast strains are commonly used as a model for studying the amyloid biology and prion propagation.

We selected 6 genes with notable insertions and deletions in the repetitive sequences encoding potentially amyloidogenic proteins: *ASG1, DDR48, GAL11, NSP1, NUP100*, and *SCH9*. We validated all structural variations in these genes by PCR and Sanger sequencing (Supplementary Figure S5, Supplementary Table S4), and evaluated the amyloidogenic potential of the protein sequences using the ArchCandy software (Ahmed *et al.* 2015). For Ddr48 and Nsp1 we found no differences in the predicted amyloidogenicity between PGC and S288C alleles (Supplementary Figure S6). For Gal11 we found a very slight decrease in amyloidogenicity in certain parts of the protein, while the amyloidogenic potential of the C-terminal region of Asg1 was significantly increased (Supplementary Figure S6). We also analyzed an influence of insertions in the mitochondrial *VAR1* gene which resulted in expansion of two poly-asparagine tracts of the respective protein (Supplementary Table S2). Both insertions occurred in amyloidogenic regions of Var1 and led to increase in its amyloidogenicity (Supplementary Figure S6).

**Figure 5:**
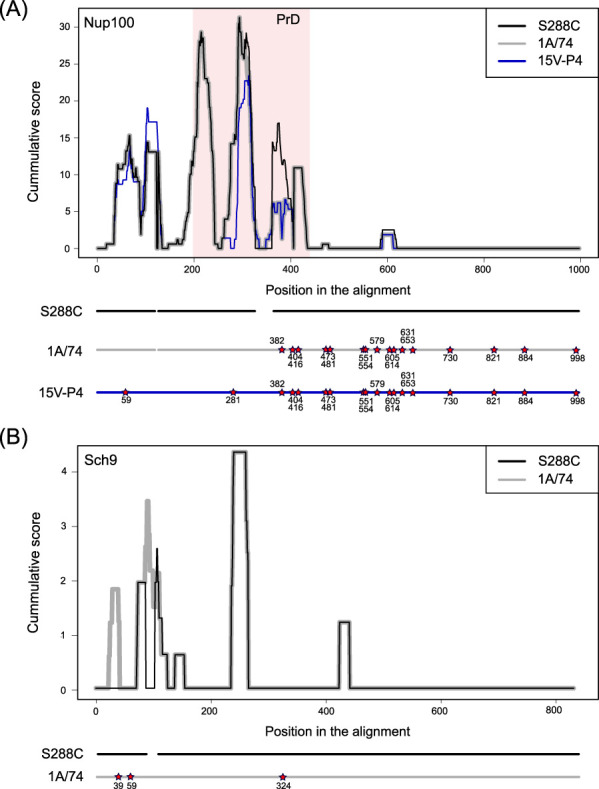
Amyloidogenicity prediction for the proteins harboring insertions or deletions in tandem repeat coding sequences. The amyloidogenic properties of the Nup100 (A) and Sch9 (B) proteins in different strains are displayed. The stars indicate significant substitutions. Coordinates of the substitutions represent positions in the alignment. Profiles were generated using ArchCandy software (Ahmed *et al.* 2015).

The most interesting example of coding SVs comes from the *NUP100* gene that encodes a Nup100 nucleoporin, a protein that is known to form the [*NUP100*^+^] prion in yeast (Halfmann *et al.* 2012). Among yeast strains two structurally distinct alleles of *NUP100* exist. The reference allele is shared by strains such as W303 and CEN.PK, and another one is present in strains D273-10B and Sigma1278b and contains multiple amino acid substitutions and two insertions, one of which resides within the prion domain of Nup100. The sequence of the *NUP100* from the ancestor Peterhof strain, 15 V-P4, derived from the previous PGC strain genomic study (Drozdova *et al.* 2016b), exemplifies an allele with two insertions (Figure 5A). We found that in both U-1A-D1628 and 74-D694 a unique chimeric allele is present, with the sequence near the N-terminal end of the protein being identical to the reference S288C allele, and the middle and C-terminal part of the protein originating from the ancestral PGC allele. The likely recombination breakpoint is located inside the prion domain. The ancestral PGC allele exhibited decreased amyloidogenic potential of the middle region of the protein (including the prion domain) and a slight increase in amyloidogenicity at the N-terminus of the protein. Remarkably, a chimeric allele of the U-1A-D1628 and 74-D694 shares the N-terminal part of the prion domain with the S288C lineage, suggesting that aggregate-forming ability of the protein is at least partially restored.

Another important example of variation in the Q/N-rich aggregation-prone protein is the insertion in the *SCH9* gene present in the PGC strains. Sch9 is a protein kinase that is a downstream component of the TORC1 signaling pathway. Apart from its native biological functions, involving regulating protein synthesis and maintenance of genome stability, Sch9 is capable of forming protein aggregates upon overproduction. At the same time, overproduction of Sch9 causes changes in cell shape, as recently shown by our laboratory (Drozdova *et al.* 2018). Furthermore, the effect of *SCH9* overexpression on cell shape is exacerbated by the deletion of its N-terminal domain that has the highest amyloidogenic potential (Figure 5B), suggesting that the N-terminal region might prevent the excessive activity of Sch9. Notably, an insertion that is present in the PGC strain further enhances the capacity of the N-terminal domain to aggregate, as demonstrated by ArchCandy analysis (Figure 5B).

Apart from amyloidogenic proteins, we also discovered a duplication of a 22 amino acid sequence in the alpha factor sequence (the *MF(ALPHA)1* gene). Similar sequence variants have been found in other strains according to the strain alignment viewer of *Saccharomyces* genome database (Song *et al.* 2016). However, it is unclear whether this variation contributes to the mating phenotype of the strains. We also validated a 7 amino acid insertion in the repetitive sequence of the G/M region of one of the two major cytosolic J-proteins, Sis1, in both 74-D694 and U-1A-D1628. This variation and its possible influence on yeast prion propagation has been described recently (Yu and King 2019). Variation in this region of the Sis1 protein is also widespread, according to the strain alignment viewer of Saccharomyces genome database (Song *et al.* 2016). Overall, frequent variation in both aggregation-prone proteins and molecular chaperones highlights the possible influence of the genotype of the strain on the formation and propagation of protein aggregates. This conclusion, in turn, predicates the need to account for the strain’s genetic background when investigating protein aggregates in yeast.

Taken together, our results further emphasize the utility of third-generation sequencing technologies for generation of complete chromosome-level assemblies of small eukaryotic genomes. We successfully constructed a chromosome-level genome assembly of two frequently used laboratory yeast strains, and conducted a detailed analysis of structural variations in these genomes. We confirm some of the major trends in the evolution of laboratory yeast strain, such as relatively fast chromosomal rearrangements at chromosome ends combined with accumulation of unbalanced SVs in the core genome, including tandem duplication of whole genes (such as *CUP1* or *ENA*) or smaller repetitive sequences (e.g., in genes encoding amyloidogenic proteins), and relocation of TEs. The latter, notably, even allows reconstructing the evolutionary relationships between the strains. Our results provide important insights into the genotype of frequently used strains from the PGC which might be taken into account when conducting research in yeast molecular biology.
